# Do Metastatic Kidney Cancer Patients Benefit From Cytoreductive Nephrectomy? A Real-World Retrospective Study From the SEER Database

**DOI:** 10.3389/fsurg.2021.716455

**Published:** 2021-08-30

**Authors:** Cheng Li, Ruiliang Wang, Wenchao Ma, Shenghua Liu, Xudong Yao

**Affiliations:** ^1^Department of Urology, Shanghai Tenth People's Hospital, Tongji University School of Medicine, Shanghai, China; ^2^Shanghai Clinical College, Anhui Medical University, Hefei, China

**Keywords:** cytoreductive nephrectomy, metastatic kidney cancer, overall survival, cancer specific survival, SEER

## Abstract

**Introduction:** The benefit of cytoreductive nephrectomy (CN) for metastatic kidney cancer has been challenged recently. The study aimed to evaluate the prognostic roles of surgical resection of primary tumor site for metastatic kidney cancer under a real-world setting.

**Methods:** The Surveillance, Epidemiology, and End Results (SEER) database (2010–2015) and the overall survival (OS) and cancer-specific survival (CSS) were evaluated using the Cox proportional hazards regression model. One-to-one matching using the propensity score was used to estimate and compare the survival rates.

**Results:** The SEER data contain records of 8,932 patients from 2010 to 2015. The data showed that 61.7% of the patients underwent CN while 38.2% did not receive any surgery. The median survival month for a patient without surgery was 4 months and for a patient with surgery was 19 months. The multivariate analysis showed that surgical resection of the primary tumor site was an independent favorable predictor for both OS and CSS (all *p* < 0.001) in the original and the matching cohort.

**Conclusions:** In the era of target therapy, CN might still be a vital method to treat metastatic kidney cancer.

## Introduction

Kidney cancer remains one of the most commonly diagnosed cancers worldwide. The estimated new cases ranked sixth in men and 10th in women, and ~15% are metastatic at diagnosis (1). Due to the absence of effective cytotoxic chemotherapy and the radioresistance to renal cell carcinoma (RCC), the prognosis of metastatic renal cell carcinoma (mRCC) is poor and the median survival rate of 1- and 2-year is approximately 10–20% (2).

The standard of care for metastatic kidney cancer has been under debate over the decades. Cytoreductive nephrectomy (CN) is used to resect the primary tumor in place and single or oligo-metastatic disease. In the era of cytokines (before 2004), the study showed that combining CN with immunotherapy improved overall survival (OS) rate compared with immunotherapy alone, which favored CN as a standard of care for patient with metastatic disease for a long time (3, 4). However, since the introduction of vascular endothelial growth factor (VEGF) target therapy in 2004, it has emerged as an important anti-tumor activity with relatively less toxicity (5, 6), making CN less important. The role of nephrectomy in treating metastatic kidney cancer has again aroused controversy.

Several meta-analysis and retrospective studies supported CN combined with target therapy instead of target therapy alone (7, 8). However, a recent randomized CARMENA study opposed the above review and demonstrated that sunitinib alone was not inferior to sunitinib plus nephrectomy for the primary end point of OS. This again questioned the place of debulking nephrectomy under the background of current tyrosine kinase inhibitor (TKI) therapy (9). Despite the newly released level I evidence, surgical resection of the primary tumor site in mRCC is still an option preferred by many urologists in daily practice.

Thus, this study aimed to investigate the controversial issue again to check whether CN brought more survival benefits compared with the conservative management of M1 kidney cancer, based on the Surveillance, Epidemiology, and End Results (SEER) database, one of the largest population-based cancer databases. This study is still significant as this topic needscontract-sponsor id="cn001" more discussion in a large real-world situation. Furthermore, we performed a 1:1 propensity score matching (PSM) analysis, which is a powerful method to minimize the selection bias, to create a 1:1 matched cohort with well-balanced baseline characteristics.

## Patients and Methods

### Study Population

**Figure 1:**
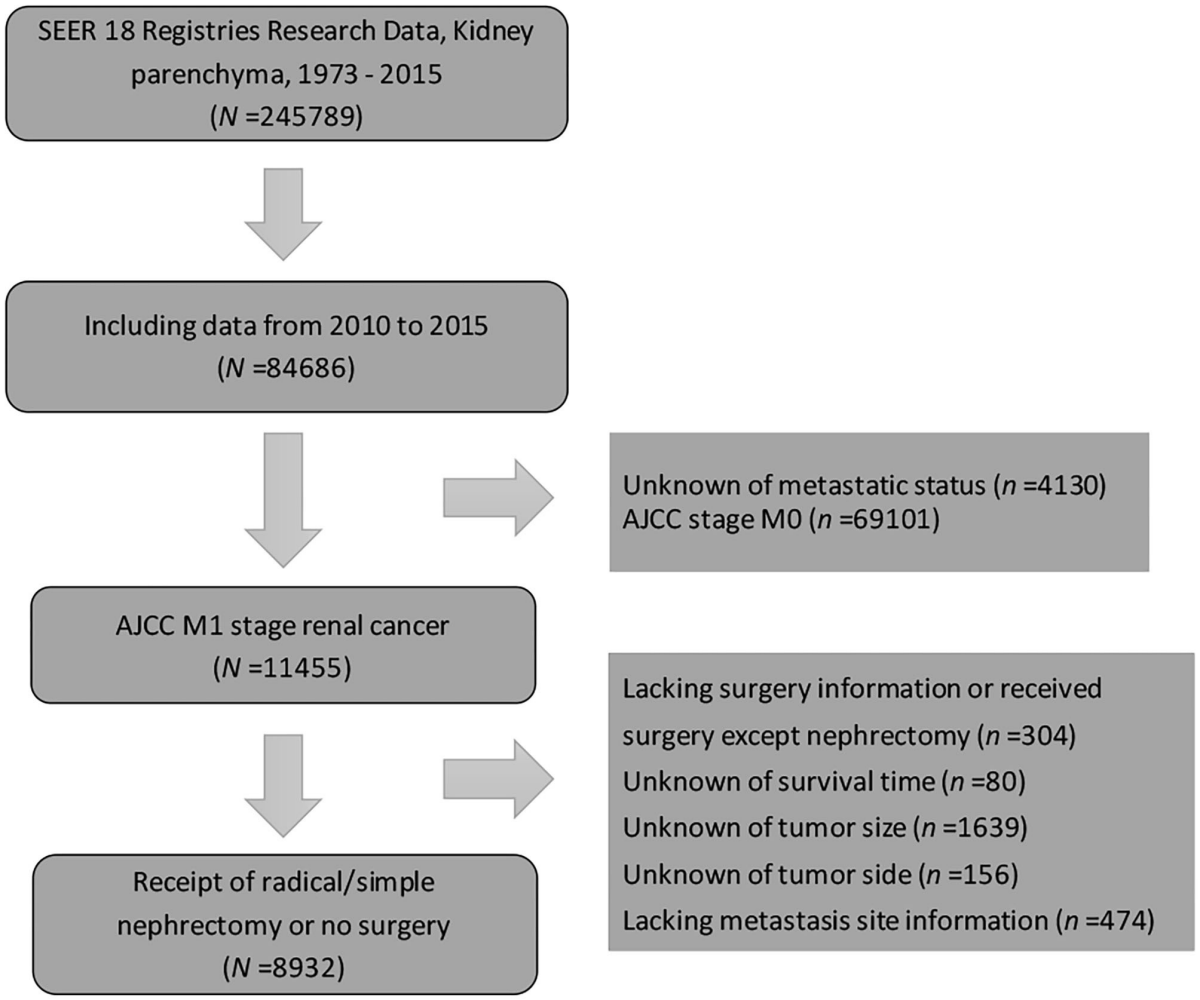
Study population diagram. Patient selection flowchart. AJCC, American Joint Committee on cancer; SEER, Surveillance, Epidemiology, and End Results.

Patients diagnosed with metastatic (M1 stage) kidney parenchyma carcinoma from 2010 to 2015 were identified from the SEER database due to the unavailability of metastasis information before 2010. Kidney parenchyma carcinoma with distant metastasis as the first primary malignancy was the inclusion criterion followed in the study. Information of the distant metastatic organs included the bones, brain, liver, and lungs. As the study aimed to investigate the impact of cytoreductive surgery on patients with kidney cancer, patients without sufficient surgery information and those who only underwent local treatment procedures (electrocautery, laser ablation, photodynamic therapy, or cryosurgery) were excluded. The current study was granted an exemption from the Ethics Review Board in the institution because the SEER program collects data from population-based cancer registries with anonymous information. It is a publicly available database with no human participants involved. Hence, no ethical approval was needed. A detailed description of patient selection is shown in Figure 1.

### Clinical Factors and Follow-Up Information

Clinical information was collected, such as demographics (age at diagnosis, gender, and year), tumor characteristics (tumor size, laterality, T stage, N stage, histology, bone metastasis status, brain metastasis status, liver metastasis status, and lung metastasis status). Follow-up information was collected, such as survival months, survival status, and cancer-specific survival (CSS) status. The major end point was overall mortality and cancer-specific mortality. The duration of OS and CSS was defined as the time from kidney carcinoma diagnosis to the date of all-cause and cancer-specific death, respectively.

### Propensity Score Matching

The propensity scores were estimated using the logistic regression model that had been established from the factors which potentially affected a decision of treatment modalities: no surgery vs. cytoreductive surgery group. Those factors were age, gender, pathologic T stage, N stage, laterality, bone metastasis, brain metastasis, liver and lung metastasis, and histology. With the estimated propensity score, a one-to-one matched cohort was constituted using the nearest-neighbor methods, within a 0.000001 caliper size.

### Statistics

A descriptive study of all the variables was conducted. The patients were divided into a group with no surgery and a group with resection of primary tumor site. Pearson's chi-square test was used to compare the categorical variables between two groups, and the *t*-test was used for continuous parametric variables. Survival curves were estimated using the Kaplan–Meier method. The log-rank test was used to assess significant differences between OS and CSS. The multivariate Cox proportional hazards regression analysis was employed to evaluate the prognostic factors in the original and matched group, and hazard ratios (HRs) along with 95% confidence interval (95% *CI*) were calculated. Only variables of significance in univariate analysis were put into the multivariate analysis. The differences were considered to be significant if *p* < 0.05. The statistical analysis was performed using IBM SPSS version 19.0 (SPSS, IBM company, Armonk, NY, USA).

## Results

### Patient Characteristics

A total of 8,932 patients with metastatic kidney parenchyma carcinoma at the time of diagnosis from 2010 to 2015 were included. The detailed patient characteristics are displayed in Table 1. In the study, only 5,518 (61.7%) patients underwent surgical resection of the primary tumor site while 3,414 (38.2%) patients did not receive any surgery. The median age of patients with no surgery and with surgery was 61 and 68 years old, respectively. The median survival month for patients without surgery was 4 months (0–71 months) and for patients with surgery, it was 19 months (0–71 months).

**Table 1:** Patients characteristics between surgery and no surgery group.

	**Original cohort**			**Matching cohort**		
	**Surgery**	**No surgery**	***p*** **-value**	**Surgery**	**No surgery**	***p*** **-value**
Age (years)			<0.001			0.889
Mean	67.84	61.11		64.18	64.07	
Median (Range)	68 (4-101)	61 (8-92)		63.92 (1-87)	63.76 (38–87)	
Gender (n%)			0.006			0.561
Male	3,707 (67.2)	2,389 (70)		245 (84.2)	250 (85.9)	
Female	1,811 (32.8)	1,025 (30)		46 (15.8)	41 (14.1)	
T Stage			<0.001			0.307
T1	1,453 (26.3)	387 (11.3)		67 (23)	68(23.4)	
T2	1,251 (22.7)	443 (13.0)		48 (16.5)	49 (16.8)	
T3	1,206 (21.9)	2,291 (67.1)		159 (54.6)	150 (51.5)	
T4	621 (11.3)	278 (8.1)		16 (5.5)	17 (5.8)	
Tx	987 (17.9)	15 (0.4)		1 (0.3)	7 (2.4)	
N Stage			<0.001			0.726
N0	2,936 (53.2)	2,345 (68.7)		242 (83.2)	240 (82.5)	
N1	1070 (19.4)	514 (15.1)		29 (8)	28 (9.6)	
N2	832 (15.1)	448 (13.1)		18 (6.2)	18 (50)	
Nx	680 (12.3)	107 (3.1)		2 (0.7)	5 (1.7)	
Tumor size			<0.001			0.935
<5	1,221 (22.1)	327 (9.6)		37 (12.7)	40 (13.7)	
5–10	2,632 (47.7)	1,611 (47.2)		146 (50.2)	144 (49.5)	
>10	1,665 (30.2)	1,476 (43.2)		108 (37.1)	107 (36.8)	
Laterality			<0.001			0.868
Right	2,750 (49.8)	1,590 (46.6)		147 (50.5)	149 (51.2)	
Left	2,727 (49.4)	1,822 (53.4)		144 (49.5)	142 (48.8)	
Both	41 (0.7)	2 (0.1)		0 (0)	0 (0)	
Bone metastasis			<0.001			0.792
Absent	3,152 (57.1)	2,394 (70.1)		193 (66.3)	196 (67.4)	
Present	2,366 (42.9)	1,020 (29.9)		98 (33.7)	95 (32.6)	
Brain metastasis			<0.001			1
Absent	4,766 (86.2)	3,167 (92.8)		279 (95.9)	279 (95.9)	
Present	752 (13.6)	247 (7.2)		12 (4.1)	12 (4.1)	
Liver metastasis			<0.001			0.874
Absent	4,062 (73.6)	3,005 (88.0)		269 (92.4)	270 (92.8)	
Present	1,456 (26.4)	409 (12.0)		22 (7.6)	21 (7.2)	
Lung metastasis			<0.001			0.786
Absent	2,017 (36.6)	1,450 (42.5)		86 (29.6)	89 (30.6)	
Present	3,501 (63.4)	1,964 (57.5)		205 (70.4)	202 (69.4)	
Histology			<0.001			0.934
ccRCC	1,574 (28.5)	2,144 (62.8)		158 (54.3)	157 (54)	
Other histological type	3944 (71.5)	1270 (37.2)		133 (45.7)	134 (46)	

*Surgery, the patients underwent cytoreductive nephrectomy; No surgery, the patients did not undergo cytoreductive nephrectomy*.

Using the propensity score, the matched cohort composed of 582 patients was constructed Table 1 with 291 patients in the surgery group and another 291 in the no surgery group. There was no significantly different distribution of clinical characteristics between the two groups in the matched cohort. The median survival month for patients without surgery was 7 months (0–67 months) and for patients with surgery was 27 months (0–71 months).

### Distribution and Prognosis of Distant Metastatic Sites

**Figure 2:**
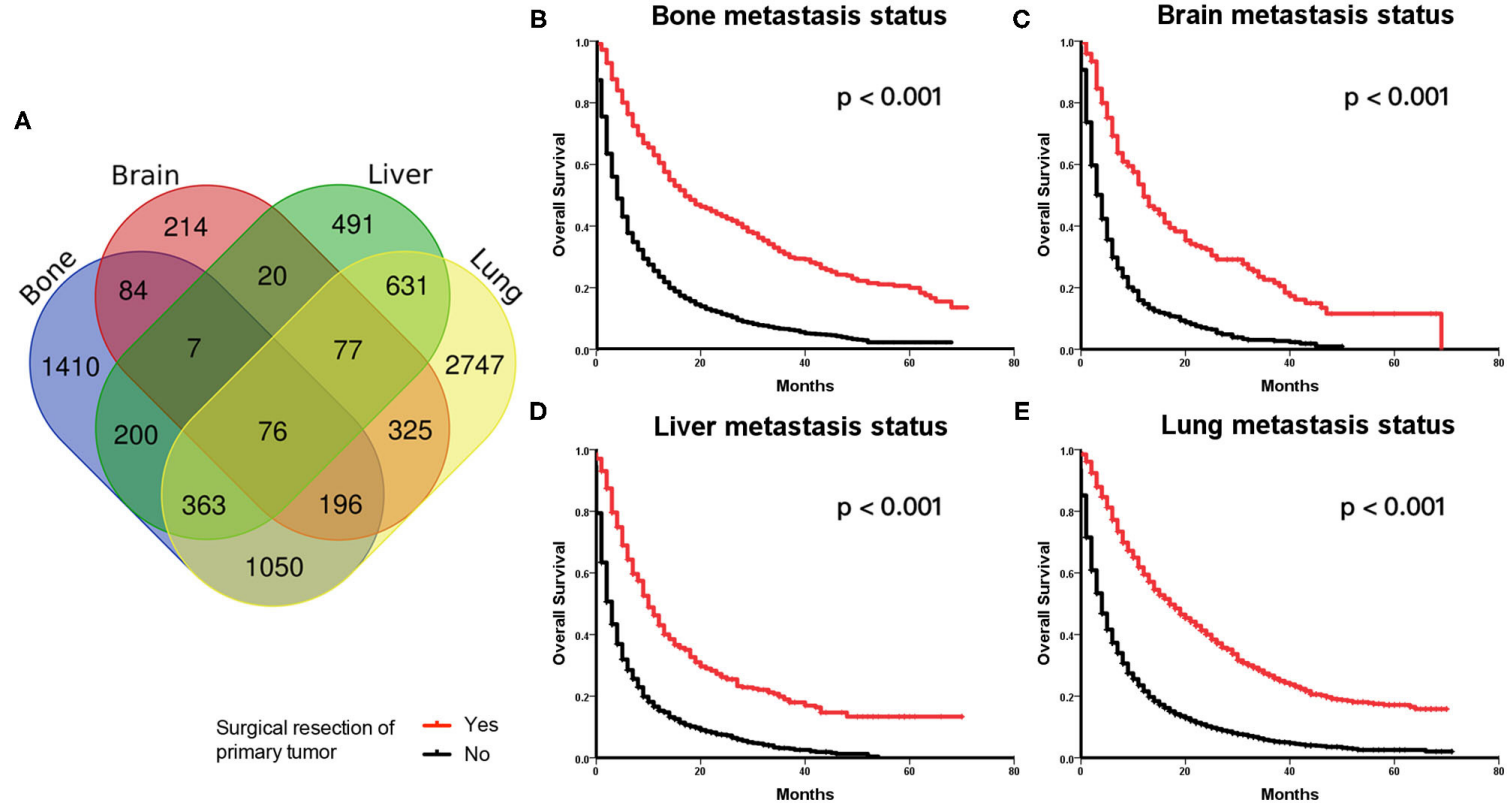
**(A)** Venn diagram of distributions of main distant metastatic organs in metastatic kidney cancer. **(B–E)** Kaplan–Meier Curves and log-rank test for the survival analysis in the original cohort. Comparing the overall survival (OS) between the surgery and no surgery groups among patients with bone metastasis status **(B)**, brain metastasis status **(C)**, liver metastasis status **(D)**, and lung metastasis status **(E)**, respectively.

The main metastatic sites of kidney cancer were the bones, brain, liver, and lungs, which composed of 7,891 (88.3%) of all patients. The information of the four main distant metastatic organs is summarized in Table 2. It was found that 5,465 (61.2%) patients were diagnosed with lung metastasis, 1,865 (22.2%) patients with liver metastasis, 3,386 (37.9%) patients with bone metastasis, and only 999 (11.2%) patients with brain metastasis. We plotted the distribution of four main metastatic organs in the Venn diagram (Figure 2A). The detailed metastatic sites of patients with more than one organ metastasis are presented in the overlapping area of the Venn diagram.

**Table 2:** Characteristics of patients and metastatic organs of original cohort.

		**Bone Metastasis**			**Brain Metastasis**			**Liver Metastasis**			**Lung Metastasis**		
	**Total**	**Absent (%)**	**Present (%)**	**p**	**Absent (%)**	**Present (%)**	**p**	**Absent (%)**	**Present (%)**	**p**	**Absent (%)**	**Present (%)**	**p**
Laterality				0.975			0.008			0.611			0.305
Right	4,340	2,696 (62.1)	1,644 (37.9)		3,830 (88.2)	510 (11.8)		3,451 (79.5)	889 (20.5)		1,663 (38.3)	2,677 (61.7)	
Left	4,549	2,824 (62.1)	1,725 (37.9)		4,070 (89.5)	479 (10.5)		3,581 (78.7)	968 (21.3)		1,791 (39.4)	2,758 (60.6)	
Both	43	26 (60.5)	17 (39.5)		33 (76.7)	10 (23.3)		35 (81.4)	8 (18.6)		13 (30.2)	30 (69.8)	
Sex				0.035			0.989			<0.001			0.011
Male	6,096	3,740 (61.4)	2,356 (38.6)		5,414 (88.8)	682 (11.2)		4,904 (80.4)	1,192 (19.6)		2,312 (37.9)	3,784 (62.1)	
Female	2,836	1,806 (63.7)	1,030 (36.3)		2,519 (88.8)	317 (11.2)		2,163 (76.3)	673 (23.7)		1,155 (40.7)	1,681 (59.3)	
T				<0.001			<0.001			<0.001			<0.001
T1	1,840	887 (48.2)	953 (51.8)		1,648 (89.6)	192 (10.4)		1,531 (83.2)	309 (16.8)		1,006 (54.7)	834 (45.3)	
T2	1,694	1,050 (62.0)	644 (38.0)		1,426 (84.2)	268 (15.8)		1,375 (81.2)	319 (18.8)		578 (34.1)	1,116 (65.9)	
T3	3,497	2,439 (69.7)	1,058 (30.3)		3,167 (90.6)	330 (9.4)		2,852 (81.6)	645 (18.4)		1,186 (33.9)	2,311 (66.1)	
T4	899	628 (69.9)	271 (30.1)		815 (90.7)	84 (9.3)		578 (64.3)	321 (35.7)		315 (35.0)	584 (65.0)	
Tx	1002	542 (54.1)	460 (45.9)		877 (87.5)	125 (12.5)		731 (73.0)	271 (27.0)		382 (38.1)	620 (61.9)	
N				0.143			<0.001			<0.001			<0.001
N0	5,281	3,281 (62.1)	2,000 (37.9)		4,647 (88.0)	634 (12.0)		4,366 (82.7)	915 (17.3)		2,146 (40.6)	3,135 (59.4)	
N1	1,584	1,005 (63.4)	579 (36.6)		1,425 (90.0)	159 (10.0)		1,159 (73.2)	425 (26.8)		578 (36.5)	1,006 (63.5)	
N2	1,280	799 (62.4)	481 (37.6)		1,173 (91.6)	107 (8.4)		958 (74.8)	322 (25.2)		484 (37.8)	796 (62.2)	
Nx	787	461 (58.6)	326 (41.4)		688 (87.4)	99 (12.6)		584 (74.2)	203 (25.8)		259 (32.9)	528 (67.1)	
Tumor Size				<0.001			0.066			<0.001			<0.001
<5	1,548	785 (50.7)	763 (49.3)		1,400 (90.4)	148 (9.6)		1,231 (79.5)	17 (20.5)		867 (56.0)	681 (44.0)	
5–10	4,243	2,515 (59.3)	1,728 (40.7)		3,745 (88.3)	498 (11.7)		3,476 (81.9)	767 (18.1)		1,704 (40.2)	2,539 (59.8)	
>10	3,141	2,246 (71.5)	895 (28.5)		2,788 (88.8)	353 (11.2)		2,360 (75.1)	781 (24.9)		896 (28.5)	2,245 (71.5)	
Histology				0.018			0.15			<0.001			0.008
ccRCC	3,718	2,362 (63.5)	1,356 (36.5)		3,281 (88.2)	437 (11.8)		3,150 (84.7)	568 (15.3)		1,383 (37.2)	2,335 (62.8)	
Other histological type	5,214	3,184 (61.1)	2,030 (38.9)		4,652 (89.2)	562 (10.8)		3,917 (75.1)	1,297 (24.9)		2,084 (40.0)	3,130 (60.0)	
Surgery				<0.001			<0.001			<0.001			<0.001
Yes	5,518	3,152 (57.1)	2,366 (42.9)		4,766 (86.4)	752 (13.6)		4,062 (73.6)	1,456 (26.4)		2,017 (36.6)	3,501 (63.4)	
No	3,414	2,394 (70.1)	1,020 (29.9)		3,167 (92.8)	247 (7.2)		3,005 (88.0)	409 (12.0)		1,450 (42.5)	1,964 (57.5)	

Those patients with different metastatic sites were extracted separately, and OS was compared between the surgery and no surgery groups. The Kaplan–Meier analysis indicated that patients receiving surgery had OS benefits among those patients with bone metastasis status (Figure 2B), brain metastasis status (Figure 2C), liver metastasis status (Figure 2D), and lung metastasis status (Figure 2E), respectively.

### Prognostic Significance of OS

**Figure 3:**
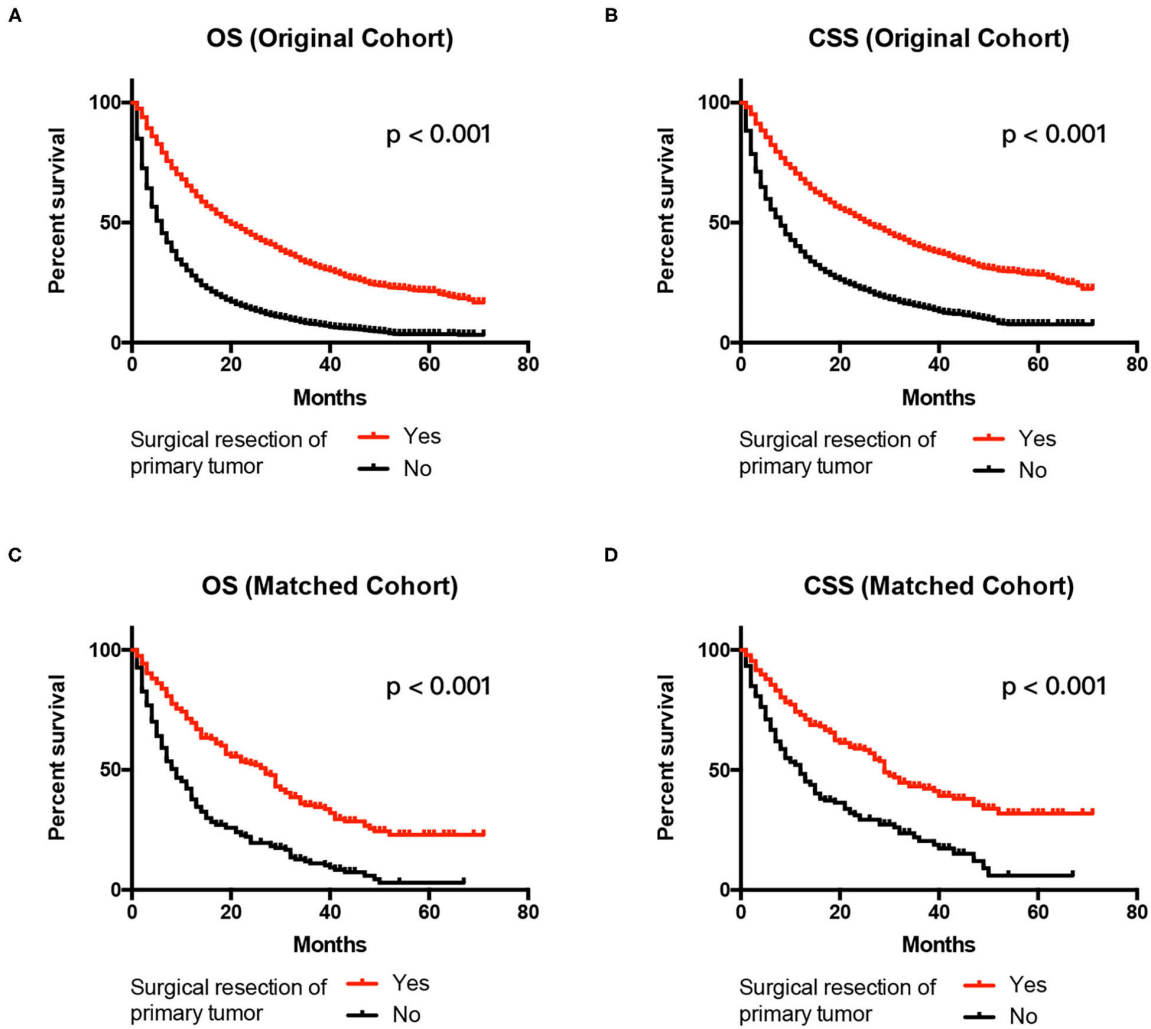
Kaplan–Meier Curves and log-rank test for the survival analysis. Comparing OS between the surgery and no surgery groups in the original cohort **(A)** and the matched cohort **(C)**. CSS was examined in the original cohort **(B)** and the matched cohort **(D)**. CSS, cancer-specific survival; OS, overall survival.

The 1-year OS rate was 68.9% for the surgery group and 38.2% for the no surgery group in the matched cohort (*P* < 0.001). The Kaplan–Meier analysis indicated that patients receiving surgery had OS benefits in both the original and the matched cohort (all *P* < 0.001) (Figures 3A,C). As shown in Table 3, patients without any surgery maintained a higher risk of mortality compared to the group receiving surgical resection of primary tumor both in the original cohort (HR 2.053, 95% *CI* 1.924–2.191, *P* < 0.001) and the matched cohort (HR 2.872, 95% *CI* 2.315–3.652, *P* < 0.001). In addition, multivariate analysis showed that older age, late T or N stage, presence of distant metastases, and non-clear cell renal cell carcinoma were all unfavorable factors associated with OS in the original cohort (all *P* < 0.001). In the matched cohort, tumor on the left side, T4 stage, brain metastasis, and non-clear cell renal cell carcinoma were linked with poorer survival.

**Table 3:** Univariate and multivariate analysis of prognostic factors influencing overall survival.

**Characteristics**	**Original cohort**				**Matching cohort**			
	**Univariate analyses**		**Multivariate analyses**		**Univariate analyses**		**Multivariate analyses**	
	**HR (95% CI)**	***P*** **-value**	**HR (95% CI)**	***P*** **-value**	**HR (95% CI)**	***P*** **-value**	**HR (95% CI)**	***P*** **-value**
**Age**	1.020 (1.018–1.022)	<0.001	**1.014 (1.012–1.106)**	**<0.001**	1.004 (0.993–1.015)	0.473		
**Gender**								
Male	Reference		Reference		Reference			
Female	1.128 (1.071–1.188)	<0.001	1.048 (0.994–1.104)	0.081	1.205 (0.918–1.582)	0.18		
**Laterality**								
Right	Reference				Reference		Reference	
Left	1.005 (0.958–1.055)	0.83			1.261 (1.031–1.532)	0.024	1.294 (1.046–1.600)	0.017
Both	1.510 (1.082–2.107)	0.015			NA	NA	NA	NA
**T stage**								
T1	Reference		Reference		Reference		Reference	
T2	1.019 (0.943–1.101)	0.627	1.082 (1.000–1.171)	0.051	0.895 (0.636–1.259)	0.523	0.830 (0.566–1.217)	0.341
T3	0.798 (0.746–0.853)	<0.001	1.169 (1.086–1.260)	<0.001	1.301 (1.01–1.676)	0.042	1.075 (0.780–1.481)	0.658
T4	1.36 (1.242–1.489)	<0.001	1.291 (1.176–1.418)	<0.001	3.104 (1.999–4.82)	<0.001	2.166 (1.258–3.731)	0.005
Tx	1.538 (1.411–1.676)	<0.001	1.159 (1.058–1.268)	0.001	1.077 (0.437–2.658)	0.872	0.656 (0.251–1.712)	0.389
**N stage**								
N0	Reference		Reference		Reference		Reference	-
N1	1.62 (1.519–1.728)	<0.001	1.363 (1.276–1.456)	<0.001	1.803 (1.31–2.48)	<0.001	1.254 (0.893–1.761)	0.192
N2	1.675 (1.563–1.796)	<0.001	1.499 (1.396–1.610)	<0.001	2.109 (1.463–3.039)	<0.001	1.422 (0.955–2.119)	0.083
Nx	1.682 (1.547–1.829)	<0.001	1.187 (1.086–1.297)	<0.001	1.326 (0.549–3.218)	0.528	2.483 (0.959–6.426)	0.061
**Tumor size**								
<5	Reference				Reference			
5–10	0.951 (0.888–1.018)	0.15			1.128 (0.826–1.542)	0.448		
>10	0.944 (0.879–1.013)	0.111			1.187 (0.862–1.636)	0.294		
**Sites of distant metastases**								
Bone -present vs. absent	1.163 (1.106–1.222)	<0.001	1.147 (1.09–1.208)	<0.001	0.791 (0.637–0.981)	0.033	0.925 (0.688–1.243)	0.603
Brain - present vs. absent	1.458 (1.355–1.569)	<0.001	1.445 (1.341–1.557)	<0.001	1.913 (1.186–3.085)	0.008	2.320 (1.394–3.862)	0.001
Liver - present vs. absent	1.761 (1.663–1.865)	<0.001	1.446 (1.363–1.533)	<0.001	1.567 (1.096–2.242)	0.014	1.460 (0.984–2.167)	0.06
Lung - present versus absent	1.243 (1.182–1.308)	<0.001	1.224 (1.161–1.29)	<0.001	1.216 (1.153–1.282)	<0.001	1.088 (0.792–1.494)	0.604
**Histology**								
ccRCC	Reference		Reference		Reference		Reference	
Other histological type	2.119 (2.012–2.231)	<0.001	1.597 (1.513–1.686)	<0.001	1.721 (1.405–2.108)	<0.001	1.820 (1.462–2.266)	<0.001
**Surgical resection of primary tumor**								
Yes	Reference		Reference		Reference		Reference	
No	2.787 (2.64–2.942)	<0.001	2.053 (1.924–2.191)	<0.001	2.506 (2.035–3.085)	<0.001	2.872 (2.315–3.562)	<0.001

*NA, not available; HR, hazard ratio; CI, confidence interval*.

### Prognostic Significance of CSS

The 1-year OS rate was 72.6% for the surgery group and 45.2% for the no surgery group in the matched cohort (*P* < 0.001). Similarly, patients receiving surgery had better CSS in both the original and the matched cohort (all *P* < 0.001) (Figures 3B,D). The patients without surgery were associated with an increased risk of mortality compared to the surgery group in the original cohort (HR 2.043, 95% CI 1.900–2.197, *P* < 0.001). The trend was also observed in the matched cohort (HR 2.611, 95% CI 2.050–3.327, *P* < 0.001). Besides, elderly patients, late T or N stage, larger tumor size present of distant metastases, and non-clear cell renal cell carcinoma had significantly decreased CSS in the original cohort in the multivariate results (all *P* < 0.001). In the matched cohort, only tumor on the left side, T4 stage, brain metastasis, and non-clear cell renal cell carcinoma were associated with poorer CSS (all *p* < 0.05) Table 4.

**Table 4:** Univariate and multivariate analysis of prognostic factors influencing cancer specific survival.

	**Original cohort**				**Matching Cohort**			
**Characteristics**	**Univariate** **analyses**		**Multivariate** **analyses**		**Univariate** **analyses**		**Multivariate** **analyses**	
	**HR (95% CI)**	***P*** **-value**	**HR (95% CI)**	***P*** **-value**	**HR (95% CI)**	***P*** **-value**	**HR (95% CI)**	***P*** **-value**
**Age**	1.01 (1.008–1.012)	<0.001	1.007 (1.004–1.009)	<0.001	0.995 (0.982–1.008)	0.419		
**Gender**								
Male	Reference		Reference		Reference			
Female	1.109 (1.046–1.176)	0.001	1.059 (0.998–1.123)	0.059	1.163 (0.851–1.588)	0.343		
**Laterality**								
Right	Reference				Reference		Reference	
Left	1.010 (0.956–1.067)	0.721			1.269 (1.010–1.594)	0.041	1.374 (1.077–1.753)	0.011
Both	1.672 (1.167–2.397)	0.005			NA	NA	NA	NA
**T stage**								
T1	Reference		Reference		Reference		Reference	
T2	1.154 (1.055–1.263)	0.002	1.035 (0.934–1.147)	0.509	1.147 (0.762–1.726)	0.511	0.923(0.563–1.514)	0.75
T3	0.936 (0.865–1.012)	0.097	1.169 (1.063–1.285)	0.001	1.766 (1.292–2.415)	<0.001	1.324(0.863–2.030)	0.198
T4	1.62 (1.462–1.795)	<0.001	1.342 (1.182–1.484)	<0.001	4.74 (2.915–7.705)	<0.001	3.332 (1.742–6.373)	<0.001
Tx	1.657 (1.497–1.833)	<0.001	1.147 (1.028–1.280)	0.014	1.785 (0.712–4.479)	0.217	1.012 (0.380–2.696)	0.981
**N stage**								
N0	Reference		Reference		Reference		Reference	-
N1	1.686 (1.568–1.813)	<0.001	1.377 (1.278–1.483)	<0.001	1.737 (1.203–2.508)	0.003	1.247 (0.838–1.856)	0.276
N2	1.803 (1.668–1.949)	<0.001	1.532 (1.415–1.659)	<0.001	2.3 (1.542–3.429)	<0.001	1.451 (0.925–2.275)	0.105
Nx	1.725 (1.569–1.896)	<0.001	1.208 (1.092–1.336)	<0.001	1.379 (0.513–3.706)	0.524	2.032 (0.703–5.878)	0.191
**Tumor size**								
<5	Reference		Reference		Reference		Reference	
5–10	1.113 (1.026–1.208)	0.01	1.238 (1.132–1.354)	<0.001	1.73 (1.145–2.612)	0.009	1.574 (0.946–2.531)	0.082
>10	1.182 (1.087–1.286)	<0.001	1.317 (1.190–1.458)	<0.001	1.856 (1.22–2.823)	0.004	1.284 (0.734–2.249)	0.381
**Sites of distant metastases**								
Bone -present vs. absent	1.213 (1.147–1.283)	<0.001	1.231 (1.161–1.305)	<0.001	0.761 (0.595–0.973)	0.03	1.105 (0.798–1.531)	0.548
Brain - present vs. absent	1.537 (1.416–1.667)	<0.001	1.477 (1.362–1.608)	<0.001	2.185 (1.313–3.636)	0.003	2.729 (1.585–4.699)	<0.001
Liver - present vs. absent	1.8 (1.687–1.920)	<0.001	1.448 (1.355–1.547)	<0.001	1.713 (1.16–2.531)	0.007	1.389 (0.901–2.139)	0.136
Lung - present vs. absent	1.316 (1.242–1.393)	<0.001	1.253 (1.180–1.331)	<0.001	1.556 (1.196–2.025)	0.001	1.262 (0.878–1.816)	0.209
**Histology**								
ccRCC	Reference		Reference		Reference		Reference	
Other histological type	2.013 (1.900–2.133)	<0.001	1.570 (1.48–1.671)	<0.001	1.612 (1.282–2.028)	<0.001	1.667 (1.296–2.143)	<0.001
**Surgical resection of primary tumor**								
Yes	Reference		Reference		Reference		Reference	
No	2.589 (2.437–2.75)	<0.001	2.043 (1.900–2.197)	<0.001	2.322 (1.837–2.934)	<0.001	2.611 (2.050–3.327)	<0.001

*NA, not available; HR, hazard ratio; CI, confidence interval*.

## Discussion

In the current study, we first demonstrate the survival advantage of CN over no surgery for the treatment of M1 kidney cancer using data from the SEER database since 2010. To overcome the selection bias in order to decide the treatment modality (CN vs. no surgery) from retrospective data, a propensity matching score was employed, which is commonly used in large observational studies.

There are various reasons for the gradual decline of CN. Although nephrectomy is a fairly safe procedure, CN in metastatic kidney cancer still remains a challenge, which is often accompanied with aggressive tumor feature, larger dimension, and severe adhesion to surrounding tissue, making cytoreductive surgery more difficult to perform and increases the risk of complications (10). Another important concern about surgery is that immediate CN often leads to a significant delay in the onset of systemic therapy, which fails to address the ultimately fatal metastatic disease, leaving its progression uncontrolled. Kutikov et al. (11) found that nearly one-third of patients with mRCC did not receive systemic treatment after CN and the most common reason was rapid postoperative disease progression. The third is the ubiquitous administration of target medicine in the recent years that lowers the status of surgery, which is strongly supported by the CARMENA trial.

However, in the present study, we found that CN still demonstrated significant OS and CSS advantages, which was in line with many previous findings (12, 13). CN provided clear survival benefits and has been considered the standard of care until recently for all patients with mRCC (14). One similar real-world cohort study based on the International Metastatic Renal Cell Carcinoma Database found that patients who received CN had better OS than those who did not (15). That is why CN, although a relatedly traumatic therapeutic method, is still accepted by many urologists and patients (16). The potential advantages of CN are promoting spontaneous regression, reducing the incidence of *de novo* metastases, or alleviating malignant symptoms (17). Theoretically speaking, removal of large tumor bulk within the kidney may reduce the potential for new aggressive biological clones to develop and thrive (18). Cytoreductive surgery could provide enough samples for the most accurate pathological evaluation, which could guide further drug choice or even new experimental treatments. If cytoreductive surgery is not intended at first, percutaneous biopsy of the primary tumor is commonly recommended to provide histological diagnosis and guide treatment decision (19). However, studies showed that biopsy has its limitations in identifying non-clear-cell histological subtype, sarcomatoid features, or Fuhrman grade (20). Therefore, the role of CN still could not be easily replaced currently.

This study showed that there is a huge survival difference between the CN and no surgery group, no matter in the original (19 vs. 4 months) nor the matched cohorts (27 vs. 7 months). In CARMENA study, the median OS was 18.4 months for sunitinib alone group vs. 13.9 months in the group starting with nephrectomy followed by sunitinib. The survival discordance could be explained by the difference between a real-world and a well-designed study. Besides, there are still patient selection bias and unbalance between the groups in the CARMENA study. Only intermediate and poor-risk patients were included in a higher proportion of advanced patients, which is the group already known to be least likely to benefit from CN. The points mentioned above might influence the interpretation of the CARMENA results (21). In the McIntosh A.G study, they observed that adverse features of final pathology, such as pT and pN stages, lymphovascular invasion, and sarcomatoid and rhabdoid dedifferentiation, were directly related to an increasing number of risk factors. At the same time, the increase of surgical blood loss, postoperative complications, and readmission rates are also associated with high risks (22). However, when 608 patient cohorts were appropriately risk stratified based on the surgical benefit, CN seemed to predict a survival benefit. The present study reflected that although TKI therapy has been widely used after the year 2010 in the United States, patients receiving CN still benefit. That is partly because under the real-world setting, there are still a large proportion of patients who do not follow rigid treatment scheme due to economic or social reasons. Some people even quit because of drug adverse event. One more reason is our cohort including patients with not only clear cell carcinoma but also non-clear cell carcinoma histology, which is not so effective for mere VEGF target therapy.

To our knowledge, there are two other similar studies utilizing the SEER database, all showed that patients with mRCC undergoing CN have improved survival rate in the era of target therapy. Conti et al. enrolling a total of 20,104 patients from 1993 to 2004, showing that median OS with CN was 19 months and outweighed 4 months without CN (23). Abern et al. analyzed 2,382 patients from 2005 to 2009, demonstrating that the patients who underwent CN had an improved 1-year survival rate (61 vs. 22%) (24). In the study, we included patients from 2010 to 2015 that could better reflect the current clinical practice. The survival data of this study were almost in line with the previous findings, showing that the outcome did not improve much over time. In Roussel's research, patients most likely to benefit from CN had oligometastatic disease and only had lung metastases (25). In the current study, however, for patients with mRCC with different metastatic sites, such as the bones, brain, liver, and lungs, the surgical group had significantly longer OS than the non-surgical group in the original cohort. Therefore, we are optimistic that most patients with mRCC can obtain a longer survival period from CN.

Nevertheless, we realized that the current study was limited by the retrospective and non-randomized design. First, although the patients were from a large national database, the systematic treatment information was lacking. There is a group of patients in both the surgical and non-surgical groups who were not eligible for systemic therapy. This could produce bias between the two groups and prevent further interpreting the impact on CN. Second, other physiological indicators related to prognoses, such as lactate dehydrogenase (LDH), serum hemoglobin, albumin, and calcium, were also unavailable in our dataset and might differ between the surgical and no surgical groups (26). Third, the role of CN nowadays could still be challenging as the therapeutic method of metastatic kidney cancer is progressing rapidly. Compared with traditional cytokine therapy, molecular targeted drugs can significantly improve the objective response rate of patients with mRCC, and prolong progress free survival and OS. Since 2006, the National Comprehensive Cancer Network (NCCN) and the European Association of Urology (EAU) have taken the molecular targeted drugs (sorafenib, sunitinib, temsirolimus, bevacizumab combined with interferon-α, palipanib, evermus, and axitinib) as first- and second-line treatments for mRCC (27, 28). The recent study demonstrated that nivolumab plus ipilimumab has shown significant OS and response rate than sunitinib alone, which enables immune checkpoint blockade to be recommended as frontline therapy in the latest guideline (29, 30). A recent real-world study showed that immune-oncology treatment was associated with a better OS in patients who had previously undergone CN (31). Therefore, with the rapid development of systemic treatment of mRCC, a more well-designed randomized study is warranted to investigate the role of CN under the new era of immune therapy. In addition, clinicians should also carefully evaluate patients using pre-operative patient risk stratification tools before offering CN (32).

In a word, the recent study again highlights the importance of surgery for patients with M1 kidney cancer. The optimal choice is to find the most suitable candidate for upfront CN, such as those with good physical condition and limited metastatic tumor burden, to avoid CN or to defer in poor risk patients (33).

## Conclusion

Despite newly-emerging pieces of evidence slashing the importance of CN in M1 kidney cancer, we showed that in the real-world setting, patients undergoing cytoreductive surgery continue to have improved OS compared to those who do not. The optimal sequencing of surgery and systemic therapy warrants further study.

## Data Availability Statement

Publicly available datasets were analyzed in this study. The datasets generated for this study are available in the SEER database (https://seer.cancer.gov/about/overview.html).

## Author Contributions

XY and SL contributed to the conception of the study. RW performed the data curation and analysis. CL and WM wrote the manuscript. WM provided constructive comments to help perform the analysis and participated in the revision of the article. All authors contributed to the article and approved the submitted version.

## Conflict of Interest

The authors declare that the research was conducted in the absence of any commercial or financial relationships that could be construed as a potential conflict of interest.

## Publisher's Note

All claims expressed in this article are solely those of the authors and do not necessarily represent those of their affiliated organizations, or those of the publisher, the editors and the reviewers. Any product that may be evaluated in this article, or claim that may be made by its manufacturer, is not guaranteed or endorsed by the publisher.
